# Reviewers in 2022

**DOI:** 10.1098/rsos.230213

**Published:** 2023-03-15

**Authors:** Wendy Hall

**Affiliations:** Editor-in-Chief

The Editors and journal administrative team of *Royal Society Open Science* would like to thank all reviewers who contributed their time by refereeing manuscripts submitted last year. We are extremely grateful for your support and expertise.

From previous feedback, we know that most reviewers value recognition of their efforts by the journal and through services such as the Web of Science Reviewer Recognition Service (formerly Publons), which is integrated with *Royal Society Open Science.*

As in previous years, to provide an opportunity for recognition, we list the names of all reviewers who have opted to be included. The list also includes a number of ‘co-reviewers’ who jointly reviewed a paper with a more senior colleague, thus not only training the next generation of reviewers but also spreading some of the workload of refereeing more equitably.

This article is made permanent and citable by its digital object identifier (DOI). We encourage you to quote your contribution in your applications for tenure and grants or other forms of research assessment. Where you have provided it, we have included your ORCID so your contribution can be unambiguously assigned to you.

On behalf of all the Editors and editorial office, thanks once again to all of our reviewers past, present and future, and we look forward to your continued support of our journal.

Aalto A
Abd Ali LI https://orcid.org/0000-0002-5948-2513
Abdallah N https://orcid.org/0000-0002-4741-7298
Abdelmohsen A https://orcid.org/0000-0001-8483-4966
abdelraoof A https://orcid.org/0000-0002-4111-9716
Abdelsalam R https://orcid.org/0000-0001-7554-3577
Abdessemed A https://orcid.org/0000-0001-5190-5991
Abeyta A
Acevedo M https://orcid.org/0000-0002-8289-1497
Acevedo-Charry O
Aczel B https://orcid.org/0000-0001-9364-4988
Adamik P
Adhikari K https://orcid.org/0000-0001-5825-4191
Adolphi N
Agarwal A https://orcid.org/0000-0001-8572-7046
Aghajamali M
Agrawal A https://orcid.org/0000-0003-0095-1220
Aguilar-Perera A
Ahman M
Aich U
Aizpurua O
Ajmal T
Akhtar MN https://orcid.org/0000-0002-0645-0796
Al Borno M https://orcid.org/0000-0003-2208-9934
Albani V
Alfieri F https://orcid.org/0000-0001-8241-7309
Ali I https://orcid.org/0000-0002-6021-1411
Allan J
Allen N
Alshareef A
Al-Tabbakh A
Altay S
Alter E
Alvarado-Ortega J https://orcid.org/0000-0002-3989-7068
Alvarez-Berrios MP
Alvarez-Idaboy JR
Aly KI
Amélineau F https://orcid.org/0000-0001-9723-5858
Amirabdollahian F
Amoruso L https://orcid.org/0000-0003-4696-2187
Andersen A
Andersen A https://orcid.org/0000-0002-3831-1707
Andrada E https://orcid.org/0000-0002-9300-4056
André J-B https://orcid.org/0000-0001-9069-447X
Andrews J
Angell C https://orcid.org/0000-0002-8773-6461
Anvari F https://orcid.org/0000-0002-5806-5654
Arbour J
Ardanuc S
Armeni K
Aron A
Arranz P
Arroyo A
Aruffo E
Ashour S
Ashraf Fahmy S https://orcid.org/0000-0003-3056-8281
Ashton N
Astegiano J https://orcid.org/0000-0003-0583-7291
Atchudan R
Authier M
Ayton P
Ayyappan A
Babarao R
Babu S
Badcock N
Bai Y
Bajer-Molnár O https://orcid.org/0000-0002-2458-4659
Bakker M
Bakkes D
Balamurugan S
Balluffi-Fry J https://orcid.org/0000-0002-2365-1055
Banas J
Banerjee A
Banerjee A
Bangal P https://orcid.org/0000-0002-1802-4196
Bank S
Bankova V
Bannerjee R
Bao G
Barabadi H
Barange D https://orcid.org/0000-0003-1279-1068
Baranger D https://orcid.org/0000-0002-6659-357X
Barati A
Barma SD
Barrett A https://orcid.org/0000-0001-8433-6828
Barrios Garcia Moar MN
Bartlett J
Bartlett M https://orcid.org/0000-0002-7391-5135
Bartoszewski R https://orcid.org/0000-0002-2864-6757
Baruah G https://orcid.org/0000-0002-5635-8437
Battista N
Becchio C https://orcid.org/0000-0002-6845-0521
Beck C https://orcid.org/0000-0003-1335-971X
Becker D https://orcid.org/0000-0003-4315-8628
Becker DJ
Beehner J
Beffara-Bret B https://orcid.org/0000-0002-0586-6650
Ben Rhaiem A https://orcid.org/0000-0002-3703-8104
Benedetti Y
Benoit J https://orcid.org/0000-0001-5378-3940
Bergman T https://orcid.org/0000-0002-9615-5001
Bertolo M
Bessi A
Best A https://orcid.org/0000-0001-6260-6516
Bhadra A https://orcid.org/0000-0002-3717-9732
Bhattacharya P
Bhattacharyya S https://orcid.org/0000-0003-3256-7688
Bickel B
Billi P
Bingyi Y https://orcid.org/0000-0002-0811-8332
Birn-Jeffery A
Bissas A
Blais C https://orcid.org/0000-0001-6055-8485
Blasco D
Bocci F https://orcid.org/0000-0003-4302-9906
bodaghi M
Bodala I
Bogaerts L
Boisgontier MP
Bolet A
Bolton K
Bonani W
Bonassi A
Bonneaud C https://orcid.org/0000-0003-2248-3288
Boothroyd L
Borduas-Dedekind N https://orcid.org/0000-0001-9302-368X
Bormashenko E https://orcid.org/0000-0003-1356-2486
Bose S https://orcid.org/0000-0002-0273-9162
Bossu C https://orcid.org/0000-0002-0458-9305
Bott L https://orcid.org/0000-0002-4926-1231
Botta F https://orcid.org/0000-0002-5681-4535
Boulton R
Bousquet G http://orcid.org/0000-0003-2008-4793
Bouter L
Bowers J https://orcid.org/0000-0003-1729-6226
Bowles J
Bowling N
Bown J
Boyle E
Brainard J
Brandt A https://orcid.org/0000-0002-2583-3530
Branney P
Brazil A
Brenninger F https://orcid.org/0000-0003-1794-0536
Breuer K
Breza M
Brito P
Bromage T https://orcid.org/0000-0002-9843-7993
Bronchal Rueda A
Brook B
Brookshire G
Brown C https://orcid.org/0000-0002-0210-1820
Brown R https://orcid.org/0000-0002-4384-775X
Brown S https://orcid.org/0000-0001-5001-525X
Buchanan E https://orcid.org/0000-0002-9689-4189
Budd G https://orcid.org/0000-0001-9007-4369
Buffetaut E https://orcid.org/0000-0001-6332-9282
Bullock S
Burkhardt E https://orcid.org/0000-0002-5128-4176
Burnham B
Burnham RE
Burridge J https://orcid.org/0000-0001-6067-4187
Burton-Chellew M https://orcid.org/0000-0002-5330-0379
Büscher T https://orcid.org/0000-0003-0639-4699
Byrd JH
Byrne H
C Lopes P https://orcid.org/0000-0001-5170-3230
Caesar L
Calderan S
Caldino U
Campbell H https://orcid.org/0000-0002-0959-1594
Campeau W https://orcid.org/0000-0001-6032-0186
Cao R
Capriles J
Cardillo A
Cardoso G https://orcid.org/0000-0001-6258-1881
Carlson K
Carpenter S
Carpentras D
Carraro L
Carrillo J https://orcid.org/0000-0003-2475-3341
Carvalho JCL
Castellote M
Castilho C
Cavallini Johansen I
Cavigliasso F https://orcid.org/0000-0002-7764-4934
Cayado P https://orcid.org/0000-0003-3703-6122
Cazzola D https://orcid.org/0000-0001-7877-6755
Ceasar SA
Cepon Robins T
Cerio DG
Chaieb L
Chamberlain R
Chamorro L
Champneys A https://orcid.org/0000-0001-7772-3686
Chan C
Chan HF https://orcid.org/0000-0002-7281-5212
Chang C-C https://orcid.org/0000-0001-9853-8949
Chappell J https://orcid.org/0000-0002-8032-2231
Chatterjee B
Chatterjee J
Chen P
Chen Z https://orcid.org/0000-0002-3500-9182
Cheney J https://orcid.org/0000-0002-9952-2612
Cheng L https://orcid.org/0000-0002-9974-2973
Cheung C-N https://orcid.org/0000-0003-2020-4679
Chiaradia A
Chilvers B
Chin J https://orcid.org/0000-0002-6573-2670
Chivite M https://orcid.org/0000-0003-1917-1523
Choi S
Choonara Y
Chopik B
Chuan-Peng H https://orcid.org/0000-0002-7503-5131
Ciarambino T
Cichocka A
Cieri R https://orcid.org/0000-0001-6905-9148
Ciesielski K
Cinelli M
Ciobanu L https://orcid.org/0000-0002-4768-5452
Civai C
Ckakraborty D
Clark C https://orcid.org/0000-0001-7943-9291
Clark H
Clark R https://orcid.org/0000-0002-1100-1856
Clark T
Clavier N
Clifton S https://orcid.org/0000-0001-5949-068X
Cmelo I
Cobbing J https://orcid.org/0000-0002-4385-524X
Cochrane B https://orcid.org/0000-0001-9681-5690
Codd J https://orcid.org/0000-0003-0211-1786
Colebank M
Colling L https://orcid.org/0000-0002-3572-7758
Colom R
Colombo M
Colson A
Columbus S https://orcid.org/0000-0003-1546-955X
Comstock D
Congedo P
Conlan A https://orcid.org/0000-0002-2593-6353
Constantine R https://orcid.org/0000-0003-3260-539X
Cook A
Cook A
Cook B
Cook D
Cooke J https://orcid.org/0000-0001-8750-6482
Coomes J https://orcid.org/0000-0002-6196-5370
Corradetti A
Correa D https://orcid.org/0000-0002-1803-6488
Cota S
Cox H https://orcid.org/0000-0001-5321-5417
Cozzolino A
Crellen T https://orcid.org/0000-0003-2934-1063
Cristofolini L https://orcid.org/0000-0002-7473-6868
Croicu M
Croy I
Curet O https://orcid.org/0000-0002-3202-6841
Cusco A https://orcid.org/0000-0002-9574-5755
Cuve H
Czaja Z
Czapanskiy M
d'Ingeo S
D'Alba L https://orcid.org/0000-0002-2478-3455
Daley M https://orcid.org/0000-0001-8584-2052
Danzelle J
Dargent F https://orcid.org/0000-0002-4510-0086
Davis B https://orcid.org/0000-0002-6121-135X
Davis T
de Barros LD
de Castro A
de Sá Martins J
de Vera P https://orcid.org/0000-0002-5645-412X
Dean M
DeBruine L https://orcid.org/0000-0002-7523-5539
Deffner D https://orcid.org/0000-0002-1649-3861
Delarue J
Delean S
Della Santina C
DeLong J https://orcid.org/0000-0003-0558-8213
Delsett L
Dennard C
Deshpande A https://orcid.org/0000-0003-2581-2737
Deutchman P
Devine RT
Dhami M
Dhanasekar M
Diessner R
Díez-Díaz V
Dinh KV
Distaso M
Dixit M
Dixon D
Dixon S https://orcid.org/0000-0002-6098-481X
Dixson B https://orcid.org/0000-0003-0911-1244
Djanian S
do Nascimento FO
Dobata S
Dobrovolny H https://orcid.org/0000-0003-3592-6770
Domínguez García V https://orcid.org/0000-0002-4591-4186
Donoghue P https://orcid.org/0000-0003-3116-7463
Doolittle E
Douady S
Dourado A
Douroumis D https://orcid.org/0000-0002-3782-0091
Douthwaite M
Drysdale T
Du J https://orcid.org/0000-0002-4309-9528
Dubey R
Dudley R https://orcid.org/0000-0003-3707-5682
Dukas R https://orcid.org/0000-0003-4533-8542
Dumontheil I
Dunfield K https://orcid.org/0000-0003-3353-6146
Dunne J
Durrant L
Dussault-Picard C
Early C https://orcid.org/0000-0003-0308-2710
Eben C https://orcid.org/0000-0001-9423-1261
Eddie C
Edgecombe G https://orcid.org/0000-0002-9591-8011
Egbedina A https://orcid.org/0000-0002-8589-3025
Eichacker L
Eidels A
Elgert C https://orcid.org/0000-0002-4609-5223
Ellis E
Elmansi H https://orcid.org/0000-0002-3953-7169
Elqayam S
Elson M
Elwakeel KZ
Engelmann J
Ensslin A
Erlandsson R https://orcid.org/0000-0002-9207-5709
Errington T https://orcid.org/0000-0002-4959-5143
Esikresnha D
Etchells P https://orcid.org/0000-0002-6642-7918
Evans S
Evans T
Everts V
Evin A https://orcid.org/0000-0003-4515-1649
Ewin T
Ewing A
Exnerova A
Falk J
Fan Y
Farina SC https://orcid.org/0000-0003-2479-1268
Fasce A
Fathy N
Fawcett T https://orcid.org/0000-0001-6337-901X
Fedele M
Feigenbaum H
Feldblum J https://orcid.org/0000-0002-7515-4632
Feldman C https://orcid.org/0000-0003-2988-3145
Feliciani C https://orcid.org/0000-0003-0718-8707
Feng J
Ferguson C https://orcid.org/0000-0003-0986-7519
Fernández-Castilla B
Fernandez-Duque E
Fernandez-Oro JM
Ferreira S
Ferreira VH https://orcid.org/0000-0001-7752-2382
Ferris D https://orcid.org/0000-0001-6373-6021
Fiddyment S https://orcid.org/0000-0002-8991-2318
Fink J
Finkelstein A https://orcid.org/0000-0003-3490-7228
Fischer S
Fitzpatrick S
Fleming A https://orcid.org/0000-0003-3301-5586
Fleming S https://orcid.org/0000-0003-0233-4891
Fletcher J
Flink LG
Foa R
Foad C https://orcid.org/0000-0003-0423-2848
Fontal A
Forbes L https://orcid.org/0000-0002-9135-3594
Forsberg A
Fortunato E
Fortunato L https://orcid.org/0000-0001-8546-9497
Frank D
Frank J
Franz J https://orcid.org/0000-0001-9523-9708
Franz M https://orcid.org/0000-0001-5111-6503
Fratini A
Fretwell P
Friberg U https://orcid.org/0000-0001-6112-9586
Friedman N https://orcid.org/0000-0002-0533-6801
Frith S
Frost R
Frouin M
Funken J
Fusaroli R
Fustin J-M https://orcid.org/0000-0002-6200-6075
Gallos L
Gambi C https://orcid.org/0000-0002-1568-7779
Gardner J
Garnier S https://orcid.org/0000-0002-3886-3974
Garrido-Vásquez P https://orcid.org/0000-0002-9561-8983
Gáspár O
Gathergood N
Gaythorpe K
Gazieva G
Gedamke J
Gems D https://orcid.org/0000-0002-6653-4676
Gendron D
Gentili PL
Georgakakos P
Georgakarakos E
George AB
Georrge JJ
Gester R
Geurts D
Ghassa S
Ghosh SK
Ghosh S
Gibb R https://orcid.org/0000-0002-0965-1649
Gillies N https://orcid.org/0000-0002-9950-609X
Girard-Buttoz C https://orcid.org/0000-0003-1742-4400
Gizynski K
Glaubitz A https://orcid.org/0000-0001-9609-3712
Glisic B
Gneezy A
Gobes M https://orcid.org/0000-0003-4085-7749
Goel G https://orcid.org/0000-0002-7656-1272
Goh HC https://orcid.org/0000-0002-9250-2205
Gokce A https://orcid.org/0000-0002-5032-7007
Gomes J
Gomez Ramirez WC https://orcid.org/0000-0002-6491-2143
Gomez M
Good D
Gordon R https://orcid.org/0000-0003-1643-6979
Gottwald S
Govender R
Gozem S
Grasser L
Grassi M
Grayfer L https://orcid.org/0000-0002-1144-4884
Greely H
Green D
Greitemeyer T
Grieves L https://orcid.org/0000-0002-6836-2177
Grillet ME
Grima R https://orcid.org/0000-0002-1266-8169
Groh M https://orcid.org/0000-0002-9029-0157
Groothuis T https://orcid.org/0000-0003-0741-2174
Grosz M
Guarneri A https://orcid.org/0000-0002-9162-2731
Guenard B
Guenther A https://orcid.org/0000-0002-2350-0185
Gustison M
Gutiérrez M
Gwenaëlle G
Haggard M
Halawa M https://orcid.org/0000-0001-6358-2691
Hall B https://orcid.org/0000-0003-0355-2946
Hall H
Hall R
Hallman TA
Hamada M https://orcid.org/0000-0001-7306-2032
Hampikian G
Han B https://orcid.org/0000-0001-9487-1090
Hanel P https://orcid.org/0000-0002-3225-1395
Haney RA
Hanley D https://orcid.org/0000-0003-0523-4335
Hanlon C
Hansford H
Hanson L
Hanzelka J
Hardwicke T https://orcid.org/0000-0001-9485-4952
Harrington K
Harrison X https://orcid.org/0000-0002-2004-3601
Harvey B
HARVEY T https://orcid.org/0000-0002-2717-7004
Haslam M https://orcid.org/0000-0001-8234-7806
Hathcock D https://orcid.org/0000-0003-4551-9239
Hawkins R https://orcid.org/0000-0001-9089-8544
Hazard L https://orcid.org/0000-0002-5135-9713
He D https://orcid.org/0000-0003-3253-654X
He J
He X
Healey P
Heck PR
Hedenström A https://orcid.org/0000-0002-1757-0945
Hedge C
Helble T
Held L https://orcid.org/0000-0002-8686-5325
Heller K-G
Hellweger F
Hemery M
Henderson A https://orcid.org/0000-0003-4384-4791
Henderson E
Herrel A
Herring S
Hertz U https://orcid.org/0000-0003-4852-3516
Heywood E
Higginson A
Hill A
Hill E https://orcid.org/0000-0002-2992-2004
Hill K https://orcid.org/0000-0002-2035-2738
Hilton J https://orcid.org/0000-0002-2787-3827
Hinch R https://orcid.org/0000-0003-3169-698X
Hines E
Ho I-C https://orcid.org/0000-0003-3906-0345
Hoadley K
Hobson C
Hochachka W
Hoffmann S https://orcid.org/0000-0001-6197-8801
Hohlbein J https://orcid.org/0000-0001-7436-2221
Holley C
Homs Dones M https://orcid.org/0000-0002-8528-8140
Hong Y
Hopkins S https://orcid.org/0000-0002-8381-0601
Houdelier C
Howell J
Howell T
Howes C https://orcid.org/0000-0002-2794-1586
Hu D https://orcid.org/0000-0002-0017-7303
Hu Z
Huang W https://orcid.org/0000-0002-3023-7610
Huang Y
Hueso J
Hulme R https://orcid.org/0000-0002-9596-7729
Huneke N
Hungnes I
Hurt CP
Hussain A
Hussein K
Ibrahim N
Iglesias S
Iosilevskii G https://orcid.org/0000-0002-4114-3214
Iqbal A
Isik T
Islam Z-ul
Itahashi S
Italiani P
Ito K
Itzchakov G https://orcid.org/0000-0003-1516-6719
Ivory J
Ja W
Jacob CR
Jakubowski KL
James A https://orcid.org/0000-0003-2859-7796
James N
Jana B
Janiga G
Janik V https://orcid.org/0000-0001-7894-0121
Janis C https://orcid.org/0000-0002-2308-0520
Jaskulski R https://orcid.org/0000-0002-2318-9323
Jeantet L https://orcid.org/0000-0001-7317-3154
Jejurikar S
Jensen K
Jensen K https://orcid.org/0000-0001-8644-1680
Jeong J
Jiang X
Jiao H
Jin Y
Johansen A https://orcid.org/0000-0002-3531-7628
Johansson ML
Jolij J
Jones B https://orcid.org/0000-0001-7777-0220
Josic K
Jozsa R
Jung J-L https://orcid.org/0000-0002-8795-8056
Kaan E
Kabir KM https://orcid.org/0000-0003-0249-5417
Kadirkamanathan V
Kadrekar R
Kahane-Rapport S
Kainz B
Kalliokoski O
Kamble A https://orcid.org/0000-0003-0875-3085
Kanda E
Kang K https://orcid.org/0000-0003-1230-3626
Kantz H
‪Kapli ‪P
Kappeler P https://orcid.org/0000-0002-4801-487X
Kara HE
Karaminis T
Kartal N
Kasmarik K
Katkar G
Kaunas R
Kause A
Kavanagh E https://orcid.org/0000-0001-7202-005X
Kawabe S
Kawakatsu M https://orcid.org/0000-0003-2072-6653
Kear B https://orcid.org/0000-0002-3128-3141
Keeler A
Kekecs Z https://orcid.org/0000-0001-9247-9781
Kelly C https://orcid.org/0000-0001-8253-357X
Kelly Y
Kenaley C http://orcid.org/0000-0003-3832-7001
Kenny A
Kenrick D
Kerber L https://orcid.org/0000-0001-8139-1493
Kerr R
Kersh M https://orcid.org/0000-0002-6039-3577
Khan BA
Khandare L
Khatib F
Khodade V
Khosronejad A
Khraishi T
Kiefer J
Kim S
Kim Y
Kirejtshuk AG
Kirkman S https://orcid.org/0000-0001-5428-7375
Kirschel A
Kittakoop P
Kittelsen KE
Klaczko L
Klein A
Klemp M
Klenova A
Klotz S
Kluger A
Knorre D https://orcid.org/0000-0001-7468-6963
Kobylkov D
Kohler M
Kok E
Kolic B
Kolomeisky A https://orcid.org/0000-0001-5677-6690
Kong X-Z
König L
Kostopoulou O
Kragel P
Kraini M
Kramer A
Krasheninnikova A https://orcid.org/0000-0001-8566-8277
Kratochwil C https://orcid.org/0000-0002-5646-3114
Kriengwatana B https://orcid.org/0000-0002-4258-883X
Krishnan S https://orcid.org/0000-0002-6466-141X
Krishnan V
Krueger J
Kryštufek B
Kulkarni R
Kültz D
Kumar A
Kumar S
Kun Á https://orcid.org/0000-0002-8409-8521
Kuper-Smith B https://orcid.org/0000-0002-2565-222X
Küpper C
Kurasaki M
Kurita H
Kurokawa S
Kuyken W
Labrousse S https://orcid.org/0000-0002-8099-3254
Lackman T
Lafferty K https://orcid.org/0000-0001-7583-4593
Lakens D https://orcid.org/0000-0002-0247-239X
Lallier F
Lamb JV
Lamers W
Langergraber K
Langley E
LaPoint S http://orcid.org/0000-0002-5499-6777
Larsen PS https://orcid.org/0000-0002-7155-5965
Lasenby A
Laurent V
Laurin M
Lavretsky P https://orcid.org/0000-0002-5904-8821
Layton-Matthews ‪
Leavell B https://orcid.org/0000-0003-4443-9309
Lebreton M
Ledesma D
Leduc A https://orcid.org/0000-0002-8471-2114
Lee A
Lee A
Lee E
Legendre L https://orcid.org/0000-0003-1343-8725
Lehman J
Leise T
Leliveld L
Lengyel A
Lenormand M https://orcid.org/0000-0001-6362-3473
Leonhardt S https://orcid.org/0000-0002-8154-9569
Lepczyk C https://orcid.org/0000-0002-5316-3159
Lessios H
Levas S
Leveneur S
Li B
Li B
Li M
Li Q https://orcid.org/0000-0002-7567-9615
Li T
Li W
Lian J
Liang Y
liao x https://orcid.org/0000-0002-8637-0146
Lieberoth A https://orcid.org/0000-0003-0214-5791
Lien Y-W
Lim T-C https://orcid.org/0000-0002-7440-7722
Limburg K https://orcid.org/0000-0003-3716-8555
Lindeman AA
Linderholm A
Lindstrom B
Lingle S
Linhart P
Linton N
Littrell S
Liu H
Liu J
Liu K
Liu X https://orcid.org/0000-0002-4422-4108
Lobbia P
Loder A
Loladze I https://orcid.org/0000-0001-7615-7803
Longrich N https://orcid.org/0000-0002-9586-8907
Louchart A https://orcid.org/0000-0003-3847-6007
Louys J https://orcid.org/0000-0001-7539-0689
Loxton NJ
Lu C
Lu K https://orcid.org/0000-0003-1370-8633
Lundh A
Lunn T https://orcid.org/0000-0003-4439-2045
Luscher A
Lush P https://orcid.org/0000-0002-0402-1699
Lutz A
Ma Y
Ma Z
Ma Z
Machala Z
MacKenzie L https://orcid.org/0000-0002-8151-0525
MacLeod N https://orcid.org/0000-0002-7169-5036
Magdy G https://orcid.org/0000-0003-0543-5065
Magnusson K
Mahalingam V
Maisey K
Maiti A
Majima Y https://orcid.org/0000-0003-3579-1546
Makagon MM
Malatesta G https://orcid.org/0000-0002-7658-2364
Malcolm P https://orcid.org/0000-0003-4110-4167
Malcolm-Smith S
Malik MI https://orcid.org/0000-0001-6942-0407
Malkowski P https://orcid.org/0000-0003-0870-438X
Mandal M
Mandanipour V
Mandl M
Maner J
Maniatis G
Manić N
Manikandan E
Manlove KR
Mann A
Mann R https://orcid.org/0000-0003-0701-1274
Manning A
Manojlovic V
Mansor M
Marchetti M
Margalida A https://orcid.org/0000-0002-7383-671X
Mari L https://orcid.org/0000-0003-1326-9992
Mariello M
Marinova R
Maritz B
Marlétaz F
Marone L
Marrable D
Marsili L
Martens A
Martin A https://orcid.org/0000-0002-2843-6928
Martin B
Martin C
Martin J
Martin-Acosta P
Martin-Duran J https://orcid.org/0000-0002-2572-1061
Martinez J-N
Maselli A
Maser R
Matejko AA
Matsui T
Mattsson C
Matyus E
May RF
Mayo ML
Mayr G https://orcid.org/0000-0001-9808-748X
Mazaris A
Mazza V https://orcid.org/0000-0002-1634-3417
Mazzone G
McClintock P
McCoy M
McFarland R https://orcid.org/0000-0001-8245-9269
McFeeters B
McGowan C
McGrady M
McGrath G
McHuron E https://orcid.org/0000-0003-3147-2628
McIntyre T https://orcid.org/0000-0001-8395-7550
McShane B
Medina Peschken J
Medrano F https://orcid.org/0000-0003-4064-5471
Meegan MJ
Mehr S https://orcid.org/0000-0002-9400-7718
Mehra A
Mehta R
Meier-Augenstein W
Meijer H https://orcid.org/0000-0001-7066-6869
Meili L
Meirelles P
Mellor D https://orcid.org/0000-0002-3125-5888
Mensinger A
Menzies M https://orcid.org/0000-0002-9907-8435
Methion S
Meyer A https://orcid.org/0000-0002-1512-5741
Michael J https://orcid.org/0000-0003-4516-196X
Michele LD
Michie S
Miellet S
Mies M https://orcid.org/0000-0001-8925-7037
Milavec M
Miles W
Militao T https://orcid.org/0000-0002-2862-1592
Miller R
Miranda JGV
Mishra BK
Miskovic-Stankovic V
Mistretta B
Mistry D
Moazen M https://orcid.org/0000-0002-9951-2975
Moghaddam AO
Mohamed H
Mohring B https://orcid.org/0000-0001-8553-4041
Moleón M
Molho C https://orcid.org/0000-0002-2846-2544
Molina CB
Molina-París C https://orcid.org/0000-0001-9828-6737
Momo F
Monarrez PM
Mondini M
Mondragon R
Monroe AP https://orcid.org/0000-0003-0934-8225
Moore H
Moore M https://orcid.org/0000-0002-8255-4247
Moore S
Moran J
Moreno-Monroy AI
Morgan A
Morgan B
Morgan T
Morris M https://orcid.org/0000-0001-9962-2982
Morsky B https://orcid.org/0000-0003-3608-1634
Mossong J https://orcid.org/0000-0003-0717-9835
Mounce R
Mourao MM
Mowles SL
Mukherjee A
Mukhopadhyay S
Mullish B https://orcid.org/0000-0001-6300-3100
Munch JC
Mundry R
Mungkasi S
Muñoz M
Murray B
Murray L https://orcid.org/0000-0002-7810-9546
Musa N
Mutabazi I
na Dutt A
Nakata T https://orcid.org/0000-0002-0378-2242
Nasrin F
Naturman C
Naude V
Naudet F https://orcid.org/0000-0003-3760-3801
Navale G
Ndlovu M https://orcid.org/0000-0002-2997-9430
Nebelsick J
Nevitt G
Newing A
Newton I
Nguyen D
Nguyen N
Nhamo L
Niedenthal P
Nielsen M
Nightingale S
Nigri A https://orcid.org/0000-0002-2707-3678
Nijenhuis Jte
Nityananda V https://orcid.org/0000-0002-2878-2425
Noguera J
Norden K
Norman D
Nowicki S https://orcid.org/0000-0002-6564-905X
Nunan D
Nuttall W
Oaknin D
Ocal K
Ocklenburg S
Odriozola JA https://orcid.org/0000-0002-8283-0459
Oh DW https://orcid.org/0000-0002-2105-3756
O'Hanlon N
Oji T https://orcid.org/0000-0002-1034-1111
Olausson H
Olivola C
Olsson-Collentine A
Onaka T
Ono K https://orcid.org/0000-0002-6349-9617
Onuferko T
Opsenica I
Osiejuk T
Ostojić L
O'Sullivan L
Otunuga O
Outeda P
Özden S
Pace DS https://orcid.org/0000-0001-5121-7080
Paes JB https://orcid.org/0000-0003-4776-4246
Pal M
Palmer C https://orcid.org/0000-0002-1058-3428
Pan L
Panda SS
Panigrahi P https://orcid.org/0000-0003-1365-5252
Paparella I https://orcid.org/0000-0002-4645-5962
Parker L
Parmar S https://orcid.org/0000-0003-0088-518X
Pasetto D https://orcid.org/0000-0001-6892-9826
Pashler H https://orcid.org/0000-0003-3644-4909
Passuni Saldana G
Pates S https://orcid.org/0000-0001-8063-9469
Patil NR
Patra P http://orcid.org/0000-0001-5700-9389
Patterson S https://orcid.org/0000-0002-5283-8919
Pedersen E
Peel L
Pei J
Peng H
Peredo C https://orcid.org/0000-0002-7217-9850
Pereira D
Perello Nieto M https://orcid.org/0000-0001-8925-424X
Permanyer I
Perrett D https://orcid.org/0000-0002-6025-0939
Peterson A https://orcid.org/0000-0002-4446-1197
Petrou E https://orcid.org/0000-0001-7811-9288
Pettijohn II TF
Pickering J https://orcid.org/0000-0002-7242-9207
Pietraszewski D https://orcid.org/0000-0002-8091-0674
Pike A https://orcid.org/0000-0003-1972-5530
Pikovsky A https://orcid.org/0000-0001-9682-7122
Pillay N
Pinfield S
Piovani L
Pirazzoli L
Plank M https://orcid.org/0000-0002-7539-3465
Pohlmann K
Poletto C https://orcid.org/0000-0002-4051-1716
Pollet I https://orcid.org/0000-0001-7207-8294
Polly P
Porffy L
Pravosudov V https://orcid.org/0000-0003-1117-7875
Preisig B https://orcid.org/0000-0002-8148-3555
Priede J https://orcid.org/0000-0003-0930-3529
Procenko O https://orcid.org/0000-0002-3929-4320
Prochazka P https://orcid.org/0000-0001-9385-4547
Prokosch ML
Przelomska N
Puddu F https://orcid.org/0000-0002-2033-5209
Punzo G
Purohit R https://orcid.org/0000-0002-2101-9398
Qiao A
Quinn T
Qureshi A
RSS
Rabajante J https://orcid.org/0000-0002-0655-0893
Rae A https://orcid.org/0000-0003-3420-4135
Ragab MAA
Raghunathan S
Rahaman A
Rahman I https://orcid.org/0000-0001-6598-6534
Raja N https://orcid.org/0000-0002-0000-3944
Rajendran V
Rallabandi R
Ramey R
Ramirez I
Ramoelo A
Rast W
Ravinetto R https://orcid.org/0000-0001-7765-2443
Rawat S
Rawson F https://orcid.org/0000-0002-4872-8928
Rayner J
Read J https://orcid.org/0000-0002-9029-5185
Reber R
Reber S
Reddin C
Reddy BV
Reddy M
Reichard D https://orcid.org/0000-0002-1219-9219
Reid A https://orcid.org/0000-0003-0511-4640
Reimer J
Reina F
Reisinger R https://orcid.org/0000-0002-8933-6875
Renkewitz F
Rhodes J https://orcid.org/0000-0001-6746-7412
Riabinina O
Richards J https://orcid.org/0000-0002-0697-2551
Richter H https://orcid.org/0000-0001-5417-8291
Rico Mancebo del Castillo Y
Riesthuis P https://orcid.org/0000-0001-6520-2453
Ristic I https://orcid.org/0000-0003-4124-4901
Ritchie S
Rivas A
Robert D
Robertson M
Robinson N https://orcid.org/0000-0001-7384-3576
Rocklöv J
Rockne R https://orcid.org/0000-0002-1557-159X
Rodó X https://orcid.org/0000-0003-4843-6180
Rodrigues F https://orcid.org/0000-0002-0145-5571
Rodríguez R https://orcid.org/0000-0003-0262-0839
Rogers L https://orcid.org/0000-0002-9956-1769
Romanovski V
Ros Velasco J https://orcid.org/0000-0003-2763-1366
Rose J https://orcid.org/0000-0003-1745-727X
Rosell J
Ross A https://orcid.org/0000-0003-0340-7407
Ross A
Rouhi A
Rowe F
Rowell J http://orcid.org/0000-0003-1102-4545
Rozhkova E
Ruan W
Rubin-Blum M
Rubio-Fernandez P https://orcid.org/0000-0003-1622-0967
Ruf T
Ruiz M
Ruktanonchai NW
Rummel J
Ruspi ML
Russo N
Rychlowska M
Rychtar J https://orcid.org/0000-0001-6600-2939
Ryder M https://orcid.org/0000-0002-1363-8148
Ryder OA
Sadeh S
Safranek D
Saha G
Saini S
Salazar S https://orcid.org/0000-0002-5437-0280
Saleh HM
Salvietti G
Samaan MA
Samadi S https://orcid.org/0000-0003-0725-9368
Samtani S
Sánchez-Cruz N https://orcid.org/0000-0003-2707-3966
Santos A
Santos CD https://orcid.org/0000-0001-5693-9795
Saracco F
Saracco JF
Sarafoglou A
Saranathan V https://orcid.org/0000-0003-4058-5093
Sarikaya MA
Saska P
Sato T https://orcid.org/0000-0002-4660-9669
Saudargiene A
Sauter T https://orcid.org/0000-0001-8225-2954
Scagliarini T
Scerif G
Schall E https://orcid.org/0000-0002-7740-5466
Scheiner S
Schellenberg G https://orcid.org/0000-0003-3681-6020
Scheumann M https://orcid.org/0000-0002-4091-9500
Schmidt A
Schmoll M
Schofield P https://orcid.org/0000-0002-5111-7263
Schoombie S https://orcid.org/0000-0002-6566-0443
Schult J
Schwarz K
Schwerdtfeger P https://orcid.org/0000-0003-4845-686X
Scofield R https://orcid.org/0000-0002-7510-6980
Scott C https://orcid.org/0000-0003-0860-4805
Scott P
Scrivener C
Scullin M
Seahra S
Seidel D
Seki Y https://orcid.org/0000-0003-2919-3048
Sekiya T
Semeraro O
Seto M https://orcid.org/0000-0003-4709-5206
Seymour R https://orcid.org/0000-0002-3395-0059
Seymoure B
Shadwick R
Shah DA
Shannon G https://orcid.org/0000-0002-5039-4904
Sharma A
Sharma P https://orcid.org/0000-0003-3890-3590
Sharp A https://orcid.org/0000-0001-5117-5335
Shea K https://orcid.org/0000-0002-7607-8248
Shehu H
Shelke N
Shepperd J https://orcid.org/0000-0002-5329-6760
Sherratt RS
Shin YS
Shinde S
Shoemaker WR
Shuker D
Shuter B
Siddiki SMAH
Siepielski A https://orcid.org/0000-0002-9864-743X
Siettos C https://orcid.org/0000-0002-9568-3355
Sigaeva T https://orcid.org/0000-0001-6764-1089
Signorell R
Silva S
Sims NA
Siniscalchi M https://orcid.org/0000-0002-1592-6549
Sitzia S
Sivakumar G
Sivamani S
Siviglia A
Skedros J
Slater D
Smith D
Smith D https://orcid.org/0000-0002-7330-4262
Smith EK
Smith E
Smith P
Snowdon C
Snyder H
Son J-J
Song N
Soni G
Sontag A https://orcid.org/0000-0002-7088-0491
Sosa S
Sotola V
Souquet L
Speakman C https://orcid.org/0000-0002-0023-518X
Speekenbrink M https://orcid.org/0000-0003-3221-1091
Spellman B
Spicer S
Spiridonov A
Spiteri C
Sridhar H https://orcid.org/0000-0003-3286-0120
Srinivasan V
Stanley E https://orcid.org/0000-0001-5257-037X
Staubwasser M
Steacy L
Steenfeldt S
Stewart A
Stien A https://orcid.org/0000-0001-8046-7337
Stojadinović B
Storks L https://orcid.org/0000-0003-3118-6234
Stover S https://orcid.org/0000-0002-2111-7887
Strait D https://orcid.org/0000-0002-3572-1663
Stritih-Peljhan N
Sudyka J
Sujith RI
Surian L
Sutin A
Suweis S
Sveegaard S
Swaminathan S
Szabo B https://orcid.org/0000-0002-3226-8621
Szabó Z
Szollosi A https://orcid.org/0000-0003-3457-542X
Szolnoki A
Tadiri C https://orcid.org/0000-0002-9305-1960
Takami Y https://orcid.org/0000-0002-6507-2115
Takashiba S
Tam K https://orcid.org/0000-0003-2197-8705
Tan J
Tan KW
Tanaka H https://orcid.org/0000-0002-1713-2734
Tanaka T
Tanalgo KC
Tang M
Tang N https://orcid.org/0000-0002-3106-6534
Taylor J
Tchernichovski O
Teitelbaum C https://orcid.org/0000-0001-5646-3184
Teixeira R
Teng S-C
Teo J https://orcid.org/0000-0001-6869-3833
Teplitskiy M
Tepole AB
Teran E https://orcid.org/0000-0001-6979-5655
Testori M https://orcid.org/0000-0001-7292-7129
Thampi S
Theofilatos A
Thibault R https://orcid.org/0000-0002-6561-3962
Thierry G https://orcid.org/0000-0003-1325-2656
Thomas A https://orcid.org/0000-0003-1239-883X
Thomas O
Thommes E
Thompson C
Thompson M
Thompson P
Thompson S
Thorne L
Tian X https://orcid.org/0000-0002-5601-2057
Tidon R
Timms L
Toelch U https://orcid.org/0000-0002-8731-3530
Tournayre O
Toyokawa W https://orcid.org/0000-0001-8558-8568
Tran HN
Trant A
Treberg J
Treiber M
Tressoldi P https://orcid.org/0000-0002-6404-0058
Tripovich J
Truong V
Tsai C-H
Tseng J https://orcid.org/0000-0001-5335-4230
Tseng Y-C https://orcid.org/0000-0001-7489-0403
Tummolini L https://orcid.org/0000-0001-7791-0457
Tupper A https://orcid.org/0000-0002-4085-3804
Tupper P https://orcid.org/0000-0002-4340-4481
Turchyn AV
Turell M
Tuyttens F
Tverskoi D
Tweedy J
Tyler C
Tzavella L https://orcid.org/0000-0002-1463-9396
Ullah I
Umberger B
Uno M
Upchurch P
Uversky V https://orcid.org/0000-0002-4037-5857
Uzundag˘ B
Vaidya T https://orcid.org/0000-0002-2264-2595
Vaikosen EN https://orcid.org/0000-0002-2413-6127
Vainikka A
Valverde S https://orcid.org/0000-0002-2150-9610
van Assen M
Van Der Hofstadt M
van der Jeugd H
van der Linden S
van der Marel A https://orcid.org/0000-0003-3942-8314
van Dongen N
Van Wassenbergh S https://orcid.org/0000-0001-5746-4621
Varón-González C https://orcid.org/0000-0002-4577-4187
Vasilievich Zyryanov V
Vedder O
Veilleux C
Veling H
Vellido A
Verbruggen S
Verheggen F
Vernouillet A https://orcid.org/0000-0003-2525-2936
Vilela ALM
Villegas NM
Villorbina G
Vizzari G https://orcid.org/0000-0002-7916-6438
Vladu IC https://orcid.org/0000-0003-3301-1112
Vogler B https://orcid.org/0000-0002-5598-5738
Voikar V
Volkoff H https://orcid.org/0000-0003-1344-3428
Voyles J https://orcid.org/0000-0002-0073-5790
Vries Jde
Vuorre M https://orcid.org/0000-0001-5052-066X
Wagle S https://orcid.org/0000-0003-2845-463X
Wagner B https://orcid.org/0000-0002-4433-2897
Wagner G https://orcid.org/0000-0003-1086-2301
Wahab Rb
Wahba MEK https://orcid.org/0000-0002-1352-0297
Walasek N https://orcid.org/0000-0003-4411-319X
Walløe L https://orcid.org/0000-0002-8919-0511
Walton G
Wang D
Wang D https://orcid.org/0000-0002-4967-524X
Wang J
Wang P https://orcid.org/0000-0003-4202-7570
Wang X https://orcid.org/0000-0001-6825-1031
Wang Z
Warrener A
Wasowicz P https://orcid.org/0000-0002-6864-6786
Wątorek M https://orcid.org/0000-0002-2131-7440
Wegener S
Wei H
Weihmann T https://orcid.org/0000-0002-6628-1816
Weingardt M
Welch K https://orcid.org/0000-0002-3283-6510
Welch KC
Wells C
Wen T-H https://orcid.org/0000-0002-9151-8336
Whitlock J https://orcid.org/0000-0002-1118-2925
Whittles L
Wichmann S https://orcid.org/0000-0002-3257-3087
Wiesner K https://orcid.org/0000-0003-2944-1988
Williams CM https://orcid.org/0000-0002-8274-8623
Williams H https://orcid.org/0000-0003-0947-6243
Wills P
Wiltshire D http://orcid.org/0000-0003-1992-6682
Wiseman A https://orcid.org/0000-0002-9575-4387
Wochner I
Wolfe J
Wolff J https://orcid.org/0000-0002-4624-8904
Wolfinger N
Wong B
Wong F https://orcid.org/0000-0002-2309-8835
Wood A https://orcid.org/0000-0003-4773-4493
Wu L
Wu Q
Wuttke A https://orcid.org/0000-0002-9579-5357
Xiao G
Xie L-H
Xie M
Xie W
Xie W
Xie X
Xie X
Xu G
Xu T
Xu ZP
Xuan L
Xue J
Yamamura T
Yanase S
Yang B
Yang H https://orcid.org/0000-0001-7490-1870
Yang K-C
Yang Y
Yang Z
Yasukawa K
Yeung SK
Yi JS
Yin Y
Yin Z
Yio M
Yon D https://orcid.org/0000-0002-5511-3884
Young J https://orcid.org/0000-0002-8671-990X
Yu L
Yu Y-X
Yu Y
Zaccagni S
Zachreson C https://orcid.org/0000-0002-0578-4049
Zakeri JA
Zakerzadeh R
Zang Z
Zare-Dorabei R
Zarnescu A https://orcid.org/0000-0002-3620-6196
Zarzeczna NJ
Zeid A https://orcid.org/0000-0001-5426-5993
Zevin J https://orcid.org/0000-0001-8929-5914
Zhan M
Zhang B https://orcid.org/0000-0003-0956-6142
Zhang H
Zhang J
Zhang J
Zhang K
Zhang L https://orcid.org/0000-0002-9586-595X
Zhao R
Zhao X
Zhou L
Zhou M
Zhou Y
Zioga I
Zoccola M https://orcid.org/0000-0002-5356-4817
Zoete V
Zuriguel I

